# Optimization of the production of roasted-nutty aroma by a newly isolated fungus *Tolypocladium inflatum* SRH81 and impact of encapsulation on its quality

**DOI:** 10.1186/s43141-022-00445-x

**Published:** 2022-11-23

**Authors:** Hoda Hanem Mohamed Fadel, Mohsen Mohamed Selim Asker, Manal Gomaa Mahmoud, Shimaa Ragab Hamed, Shereen Nazeh Lotfy

**Affiliations:** 1 Chemistry of Flavour & Aroma Department, Food Industries and Nutrition Research Institute, National Research Centre, Dokki, Cairo, Egypt; 2 Microbial Biotechnology Department, Biotechnology Research Institute, National Research Centre, Dokki, Cairo, Egypt

**Keywords:** Process flavor, Microorganisms, GC-MS, Sensory evaluation, Nutty-like flavor

## Abstract

**Background:**

Pyrazines are used in food industry to impart the foods nutty-roasted flavor. However, their extraction from natural sources is difficult and expensive. At the same time, there is awareness against the chemical food additives. Microorganisms are approved as natural producers of flavors. The aim of the present study was to assess the ability of the newly isolated fungus *Tolypocladium inflatum* SRH81 to produce pyrazines and studying the effect of encapsulation in gum Arabic on the quality of the biogenerated volatiles. The parameters affecting the biogeneration of pyrazines were optimized. The headspace volatiles of each culture were isolated and identified by solid phase microextraction (HS-SPME) and gas chromatography coupled with mass spectrometry (GC-MS). The volatiles showed the highest pyrazines content and best nutty-roasty flavor was subjected to encapsulation.

**Results:**

The selected fungus was identified as *Tolypocladium inflatum* SRH81. A high correlation was found between the consumed sugar and dry matter content of each culture. Incubation of the fungus culture enriched with 0.5 g amino acids/50 mL medium for 12 days at pH 8 showed the highest generation of pyrazines and best odor sensory quality. Nine pyrazines were identified among them 2-methylpyrazine was the major compound after incubation for 12 days. A positive correlation was found between the total pyrazines and intensity of roasty-nutty aroma. Encapsulation gave rise to a significant decrease in the total volatiles, while the odor intensity showed insignificant decrease.

**Conclusions:**

The results of the present study revealed the potential ability of *Tolypocladium inflatum* SRH81, that was isolated from Egyptian soil, to produce pyrazines having roasted- nutty aroma.

**Supplementary Information:**

The online version contains supplementary material available at 10.1186/s43141-022-00445-x.

## Background

Pyrazines are found in wide varieties of food stuff especially roasted, fried, and grilled foods. Recently, food industry gives a great attention to pyrazines as important ingredients in raw and roasted foods [1]. Generally, pyrazines are described as nutty and roasty, depending on the alkyl substituent [2]. Due to their aromatic properties, pyrazines have many uses in flavor industry. They are commonly used as flavoring agents in numerous products, for example 2,3-dimethylpyrazine is used in gravies, beverages, and sweets, 2,5-dimethylpyrazines in breakfast cereals, 2,6-dimethylpyrazine in meals as roasted flavor [1], 2-ethyl-3-methylpyrazine in peanut products, popcorn, and bread. Pyrazines can be synthesized by chemical methods. However, recently trends in food industry are to move from chemically synthesized flavor compounds. At the same time, extraction from natural sources does not meet the growing demand for natural pyrazines, besides it is also difficult and expensive. Flavor can be synthesized by microorganisms as secondary metabolites during fermentation on nutrients such as sugar and amino acids. Precursors or intermediates can be added to the culture medium in order to promote the biosynthesis of specific flavor [2]. The use of microorganisms for production of pyrazines could thus be considered as an attractive alternative with several advantages [1].

The Food and Drug Administration (FDA) and European Food Safe Authority (EFSA) declared certain microorganisms as producers of natural and safe flavors. This gives a chance for a better economic feasibility, controlled reaction conditions and high specificity compared to extraction of flavor from natural sources [3]. Several microorganisms, including bacteria and fungi, are currently known for their ability to synthesis different alkylated pyrazines [4, 5]. Production of alkyl pyrazines by *Bacillus* strains has been studied extensively [2, 6]. The bioproduction of 2,5-dimethylpyrazine and tetramethylpyrazine was optimized by enrichment of the medium with L-threonine and acetoin, respectively [7]. Different alkylated pyrazines were detected in the headspace of *Bacillus cereus* medium [2]. High production of tetramethylpyrazine was obtained by *Bacillus subtilis* under stable pH, good oxygen supply, and favorable incubation temperature [8]. Leelawiwat et al. [9] investigated the effect of various C-sources and initial pH values of the medium on pyrazine production from newly isolated *Bacillus* sp.

Bioproduction of methylpyrazine, dimethylpyrazines, trimethylpyrazine, and tetramethylpyrazine by *Corynebacterium glutamicum* (*C. glutamicum*) was related to the metabolism of branched-chain amino acids [1]. In previous study [10], the ability of *C. glutamicum* to produce nutty like flavor from enzymatically hydrolyzed soybean protein was investigated. The perceived nutty flavor was correlated to the high content of tetramethylpyrazine. In addition to bacteria, that are the most prominent producers of pyrazines, fungi, especially *Aspergillus* and *Penicillium* are able to synthesize these compounds. *Penicillium* species found naturally in different types of cheese, are known to produce pyrazine compounds [4]. Methylpyrazine and 2, 5-dimethylpyrazine were detected in fungistatic soils [11]. Recently, Lindsay et al. [12] investigated four filamentous fungi for their potential to produce volatiles, only *Aspergillus Niger* showed the ability to produce 2, 5-dimethylpyrazine.

It is difficult and expensive to handle the liquid flavorings in food industry. Microencapsulation is an attractive approach to transfer the liquid flavorings into stable free flavoring powders that can easily handle and incorporate into dry foods. Microencapsulation protects the flavors against degradation and enhances its stability. Spray drying is the most common and economical technique used to carry out encapsulation [13].

The main aim of the present study was to evaluate the ability of fungi, newly isolated from Egyptian soil, to produce roasted- nutty flavor. The fungus that showed promising results was selected and subjected to morphological and biochemical tests and was identified by 18S rRNA method. The parameters involved in pyrazines bioproduction; incubation time, initial pH, and enrichment with amino acids, were optimized. The released volatiles were subjected to odor sensory analysis, solid phase microextraction, and gas chromatography-mass spectrometry (GC-MS) analysis. The sample showed the optimum release of the pyrazines was encapsulated in gum Arabic to produce easily handle flavouring compounds. The retention of the encapsulated volatiles was evaluated.

## Materials and methods

### Materials

Amino acids (lysine, threonine, and serine), standard compounds and standard n-paraffin (C_8_−C_22_) were purchased from Sigma Aldrich Chemical Co. (St. Louis, MO, USA). Gum Arabic, agar, maltose, glucose, (NH_4_)_2_SO_4_, KH_2_PO_4_, Na_2_HPO_4_•7H_2_O, MgSO_4_•7H_2_O, CaCl_2_•2H_2_O, FeCl_3_•6H_2_O, and ZnSO_4_•7H_2_O were purchased from Merck Company, Germany. Malt extract and peptone were purchased from Loba Chemie, Bombay, India. All other chemicals were of analytical grade.

### Microorganisms, collection, and identification

Soil samples were collected from patches free from roots and the dilution-plate method was used to isolate fungi. The suspension was diluted by sterile distilled water, shaken for 10 min and transferred into Czapek’s agar Petri dish. The Petri dishes were incubated at 28 °C for 7 days. The developing colonies were isolated and grown again for 3-times until purification, the purified colonies were used. The isolate that showed a promising roasted nutty-flavor by odor sensory analysis (showed below) was distinguished depending on morphological, physiological, and biochemical characteristics of the potential producer as determined by applying various methods [14, 15]. For further investigation, growing fungal species were maintained on slants of Czapek’s agar medium at 28 °C for 24 h. Conformation of the promising isolate was identified based on 18S rRNA gene using ITS4 primer [16]. Sequencing products were resolute on an Applied Biosystems (Foster City, CA, USA) model 3730 XL, automated DNA sequencing system. The 18S rRNA gene sequence was aligned using BLAST available at NCBI database http://www.ncbi.nlm.nih.gov/BLAST. The phylogenetic tree was constructed using the software MEGA7.

### Production medium and batch culture of fungi

The fungus was grown on malt extract agar (MA) medium (malt extract, 30; peptone, 5; agar, 15 g/L, pH, 7.0) for 7 days at 28 °C. The slant from MA medium was suspended in sterile distilled water followed by gently agitation and used as a subculture. The resulting subculture was used to inoculate (6%, v/v, 1.205 × 10^5^ spore/mL) the production medium, which contain (g/L); 30.0 maltose, 0.94 (NH_4_)_2_SO_4_, 7.0 KH_2_PO_4_, 2.0 Na_2_HPO_4_•7H_2_O, 1.5 MgSO_4_•7H_2_O, 0.008 CaCl_2_•2H_2_O, 0.008 FeCl_3_•6H_2_O, 0.0001 ZnSO_4_•7H_2_O [17], at pH 7.0 was incubated for 7 days on incubation shaker (150 rpm) at 28°C.

### Effect of incubation time, pH, and amino acid concentration

Effect of the parameters affecting the culture growth and flavor production (time, pH, and amino acids %) was estimated (Supplementary Table S1). First, the fermentation was conducted at variable incubation time (6, 9, 12, and 15 days), constant pH value 7 and without enrichment of the culture with amino acids. Then the fermentation was performed at the incubation time that showed optimal intensity of roasted-nutty aroma (shown below) and variable pH values (6, 7, 8, and 9). The effect of amino acids addition (0.5, 1.0, 1.5, and 2.0% sterile blend of theronine, serine, and lysine at ratio 1,2,2, respectively) was estimated at the time and pH values that showed the optimal results. All the experiments were conducted in 250 mL conical flask containing 50 mL of basal medium.

### Determination of reducing sugars

Reducing sugars was determined in filtrate according to DNS method [18] using glucose as a standard.

### Biomass determination

Growth was measured as a dry weight of mycelium after the desired incubation period and other culture parameters. The mycelium of each flask was filtered by Whatman filter paper (No. 1; 15 cm diameter), washed three times with distilled water and dried at 85 °C for 24 h.

### Odor sensory analysis

Evaluation of the odor quality of each culture was carried out by panelists (10 male − 10 female). The panelists were trained for 3 h to identify and define the intensity of nutty-roasted aroma in terms of appropriate reference sample (roasted peanut). The panelists separately scored the intensity of each biogenerated flavor on a category scale 0.0 (not perceptible) to 10.0 (strongly perceptible). The evaluation was carried in triplicate.

### Encapsulation roasted-nutty flavor

The fermented media of the sample showed the best results was cooled in an ice bath and filtered. Arabic gum at concentration of 10% was dispersed in filtrate, vigorously homogenized (10000 rpm/3 min) at 25 °C and spray dried by Buchi, B-290 model mini spray dryer, equipped with 0.5 mm diameter nozzle, under the conditions described in previous study [10].

### Isolation and characterization of headspace volatiles

The filtrate of each media was placed in a 100-ml headspace glass vial sealed with a PTFE faced silicon septum (Supelco, Bellefonte, PA, USA). The headspace volatiles of each filtrate and the encapsulated sample were extracted by solid phase microextraction (SPME) (Supleco, 57348-U, Bellefonte, PA, USA) with coated fiber of divinylbenzene/carboxen/polydimethyl siloxane (DVB/CAR/PDMS) (coating thickness 50/30 μm). The extraction conditions (time and temperature) were optimized [19]. The SPME was conducted according to Fadel et al. [10].

A gas chromatography (Agilent 8890 GC system) coupled to a mass spectrometer (Agilent 5977B GC/MSD) was used for analysis of the volatile compounds. The injection was conducted in the splitless mode for 5 min at 250 °C. The GC was equipped with a fused silica capillary column DB5 (60 m × 0.32 mm i.d. × 0.25 μm film thickness). The oven temperature was maintained at 50 °C for 5 min, then programmed from 50 to 220 °C at a rate of 4 °C/min, and from 220 to 280 at a rate 10 °C/min. Helium was used as the carrier gas, at flow rate of 1.1 ml/min. Mass spectra in the electron impact mode (EI) were obtained at 70 eV and scan m/z range from 39 to 400 amu. The volatile flavor compounds were identified according to NIST library of mass spectra and retention indices (KI). The amount of each individual separated compound was expressed as total ion chromatograms (TIC).

### Statistical analysis

Analysis of all tests was performed in triplicate. The data of each sample were expressed as mean ± standard deviation. The significant difference within samples were compared using one way analysis of variance (ANOVA) followed by the multiple range test L.S.D. (Duncan multiple rang test) at the significant level at *p <* 0.05.

## Results and discussion

### Screening for roasted-nutty aroma production by fungi

The primary results obtained in our study showed that there are nine isolates belong to four different species of fungi (*Penicillium*, *Aspergillus*, *Fusarium*, and *Tolypocladium*) isolated from Egyptian soil containing decayed plant litters. Each individual isolate was cultivated on Czapek’s medium to test the ability of this strain to produce roasted-nutty flavor (according to odor sensory analysis). The results exhibited that the newly isolated fungi *Tolypocladium inflatum* was the only one that produced roasted-nutty flavor and therefore was selected for further identification.

### Morphological and molecular identification of Tolypocladium inflatum

**Fig. 1:**
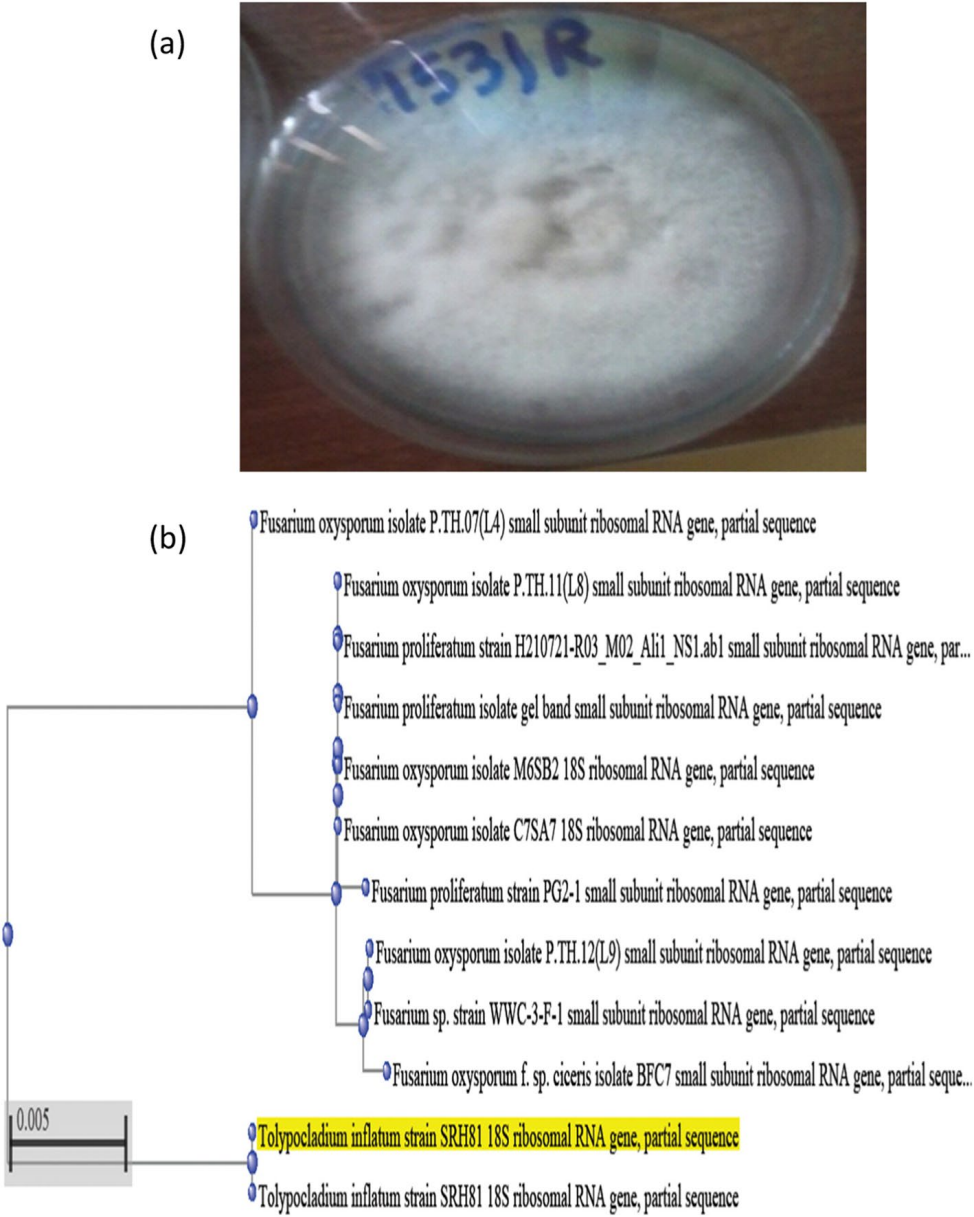
Colony morphology on potato Czapek's agar (PDA) media (**a**) and Neighbor-joining phylogenetic tree (**b**) of *Tolypocladium inflatum*

The selected isolate was identified as *T. inflatum*. *Tolypocladium* species are usually slow in growth on Czapek’s media form white colonies with a large amount of spores (Fig. 1a). Conical cylindrical was pods with terminal and lateral phialides, ellipsoid to subglobose basal and narrowing to cylindrical neck. Often crooked and bearing subglobose conidia as described in Gams [20] and Bissett [21]. The phylogenetic tree was organized in the manner that depends on the 18S rRNA sequences. The 18S rRNA gene of the *T. inflatum* was enzymatically amplified by Taq DNA polymerase. The phylogenetic analysis of sequence of *T. inflatum* SRH81 was shown in Fig. 1b with the watch closely related strains from the database. This strain deposited in GenBank under an accession number KX011178.

### Culture growth

**Fig. 2:**
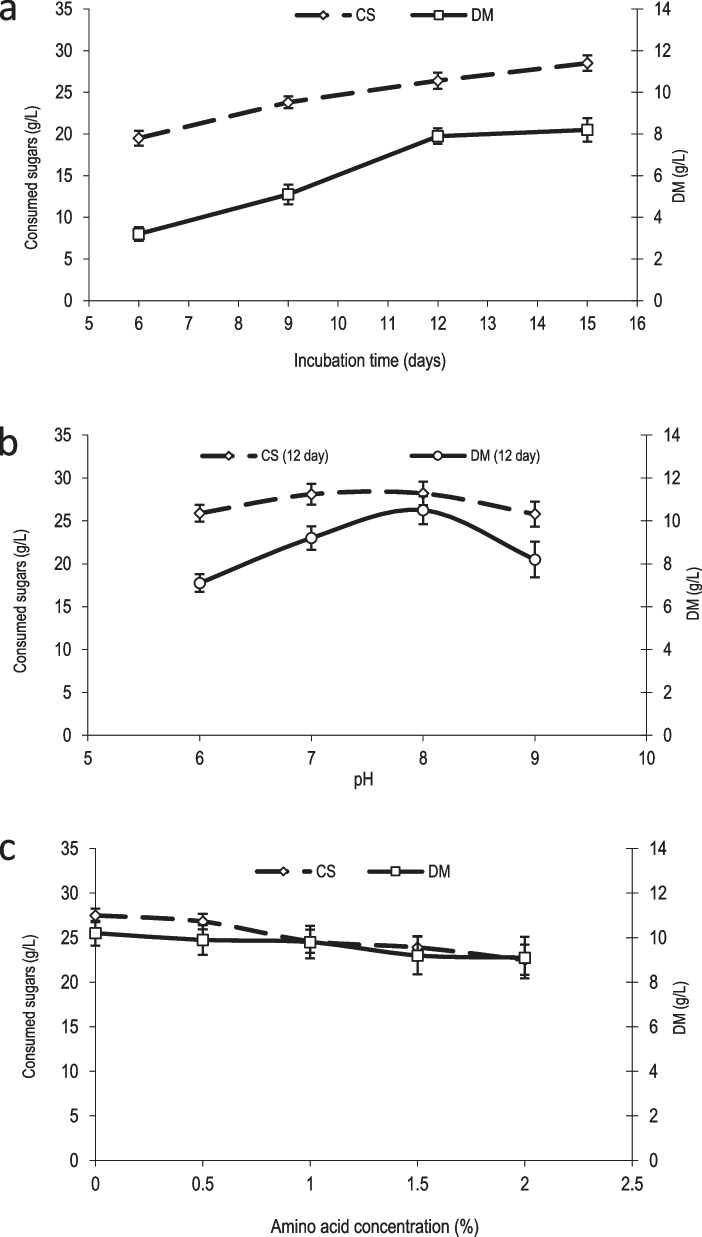
Effect of incubation times, pH values and amino acids concentration on culture growth. **a**: Incubation times (6, 9, 12 and 15 days) at pH 7 without amino acids addition; **b**: pH values (6, 7, 8 and 9) for 12 days without amino acids addition; **c**: Amino acid concentrations (0.5, 1.0, 1.5 and 2) at pH 8 for 12 days ; CS: Consumed sugar, DM: Dry matter

In the present study, the effect of different parameters (incubation time, pH values and concentration of added amino acids) on the production of roasted-nutty flavor and culture growth was investigated (as mentioned above, Supplementary Table S1). Figure 2a shows the correlation between the consumed sugar (28.5 g/L) and dry matter (8.2 g/L) during incubation for 15 days. The results revealed a positive correlation between the dry matter content and consumed sugar content during incubation time. This finding is in agreement with previous study [6]. Effect of varying the pH of the culture media at 12 days of the incubation period on the consumed sugar and dry matter content is illustrated in Fig. 2b. It is obvious from Fig. 2c that addition of variable concentration of amino acids gave rise to a noticeable decrease in the content of consumed sugar whereas no variation was detected in the dry matter content. Previous study Hernandez-Orte et al. [22] stated that addition of amino acids gave rise to a decrease in consumed sugar. Sugar catabolism resulted in accumulation of acetoin that is considered as the main precursor of alkylated pyrazines [23].

### Headspace volatiles

**Fig. 3:**
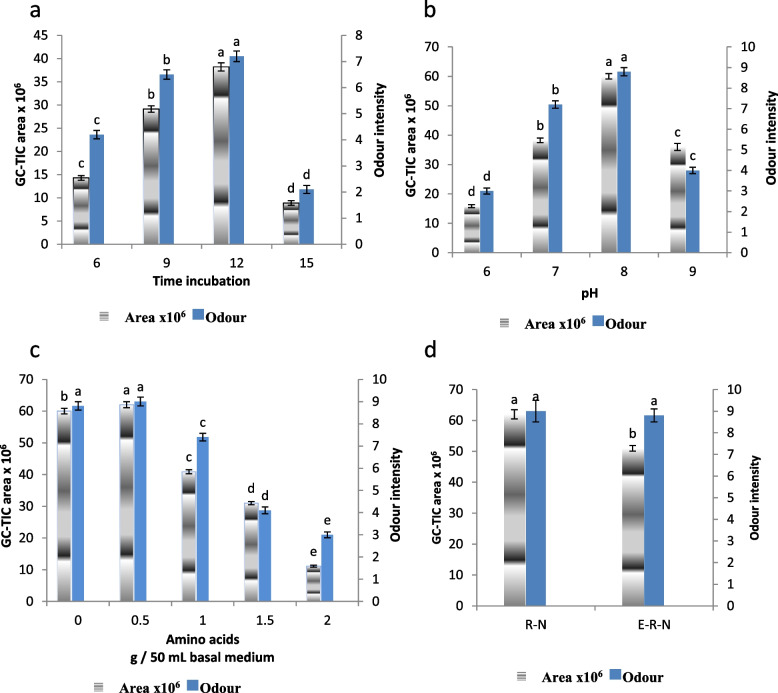
Effect of incubation times (**a**), pH values (**b**), enrichment with amino acids (**c**) and encapsulation (**d**) on total content and odour intensity of the biogenerated volatiles. R-N: Roasted-nutty flavour; E-R-N: Encapsulated roasted-nutty flavour

The metabolic production of alkylated pyrazines in microorganism cultures includes synthesis and degradation reactions that are highly dependent on culturing conditions [24]. Pyrazines are the most contributors of the desirable roasty-nutty aroma of food products [1]. Sensory evaluation of the perceived roasty-nutty aroma produced by *Tolypocladium inflatum* SRH81 during incubation for 3, 6, 9, 12, and 15 days was conducted by 10 panelists (Fig. 3). The desired aroma was detected after 6 days, followed by a gradual increase up to 12 days, where it showed the highest score of the generated roasted-nutty aroma. Incubation for 15 days revealed a significant decrease in the aroma intensity. To explore the correlation between the content of pyrazines and aroma intensity, the headspace volatiles of the *Tolypocladium inflatum* SRH81 culture at each incubation time were extracted by HS-SPEM and subjected to GC-MS analysis.

The total ion chromatograms of the separated compounds are cited in Table 1. Ten volatile compounds were identified including; acetoin (1), 2-methylpyrazine (2), 2,5-dimethylpyrazine (3), 2,6-dimethylpyrazine (4), 2-ethyl-3-methylpyrazine (5), 2,3,5-trimethylpyrazine (6), 2-acetylpyrazine (7), 2-ethyl-3,5-dimethylpyrazine (8), 3-ethyl-2,5-dimethylpyrazine (9) and 2,3,5,6- tetramethylpyrazine (10).

**Table 1:** Volatile compounds identified in headspace of *Tolypocladium inflatum* SRH81 culture during incubation

Peak no.	KI^a^	Volatile compounds^b^	Incubation time (days) Peak area^c^ × 10^6^				Description^d^
			6	9	12	15	
1	762	Acetoin	1.82^a^ ± 0.23	0.90^b^ ± 0.11	0.44^c^ ± 0.06	–	Buttry^1^
2	825	2-Methylpyrazine	10.99^c^ ± 1.28	14.36^b^ ± 1.81	19.20^a^ ± 2.43	2.65^d^ ± 0.33	Nutty green^2^
3	908	2,5-Dimethylpyrazine	0.02^d^ ± 0.00	0.11^c^ ± 0.02	0.78^b^ ± 0.10	2.06^a^ ± 0.26	Nutty^2^
4	918	2,6-Dimethylpyrazine	0.01^c^ ± 0.00	0.04^c^ ± 0.01	0.58^b^ ± 0.07	1.06^a^ ± 0.13	Nutty, roasted^3^
5	993	2-Ethyl-3-methylpyrazine	0.06^c^ ± 0.01	4.88^b^ ± 0.616	6.27^a^ ± 0.79	0.92^c^ ± 0.12	Nutty, earthy^4^
6	1000	2,3,5-Trimethylpyrazine	0.07^c^ ± 0.01	3.86^b^ ± 0.49	7.30^a^ ± 0.92	0.70^c^ ± 0.09	Chocolate^2^
7	1032	2-Acetylpyrazine	0.77^b^ ± 0.10	3.85^a^ ± 0.08	0.36^c^ ± 0.05	0.30^d^ ± 0.04	Popcorn^5^
8	1077	2-Ethyl-3,5-dimethylpyrazine	0.34^b^ ± 0.04	0.48^b^ ± 0.06	0.66^a^ ± 0.08	0.40^b^ ± 0.05	Roasty^6^
9	1080	3-Ethyl-2,5-dimethylpyrazine	0.01^c^ ± 0.00	0.04^b^ ± 0.01	0.14^a^ ± 0.02	–	Earthy-roasty^2^
10	1084	2,3,5,6-Tetramethylpyrazine	0.20^c^ ± 0.03	0.61^c^ ± 0.08	2.48^a^ ± 0.31	0.80^b^ ± 0.10	Roasted nutty^7^
		Total	14.29^c^ ± 1.81	29.13^b^ ± 3.68	38.21^a^ ± 4.83	8.89^c^ ± 1.12	

^a^Retention indices of volatile compounds on DB-5 column

^b^Compounds listed according to their elution on DB-5 column

^c^Values × 10^6^, expressed as total ion chromatograms(TIC) of GC-MS (*n* = 3) ± standard deviation

^d^Volatile compound description was performed according to^1^ Forster et al. [25], ^2^Adams and De-Kimpe [2], ^3^Bonvechi [26],^4^ Frederick et al. [1], ^5^Muller and Rappert [24], ^6^Yan et a1 [27]., ^7^Afoakwa et al. [28]. Mean values in the same row for each culture followed by different superscript lower case letters are significantly different at *P* < 0.05

The presence of acetoin with pyrazines in the headspace volatiles of *Tolypocladium inflatum* SRH81 culture suggests that it acts as a precursor of pyrazines bioproduction [6]. The metabolic activities of the microorganisms generate various precursors such as, acetolactate, acetoin, ammonia, and amino acids that are converted to pyrazines [2] by nonenzymatic reaction. In present study, the presence of the alkylated pyrazines in the headspace of the cultures could confirm the catalytic ability of the fungus to produce pyrazines.

The presence of methyl-, dimethyl-, trimethy-, tetramethyl, and mixed methyl-ethyl pyrazines have been considered to be important for flavor and aroma [1] among these compounds 2,3,5-trimethylpyrazine (TMP), 2,5-dimethylpyrazine (2,5-DMP), 2,6-dimethylpyrazine (2,6-DMP), and 2,3,5,6-tetramethylpyrazine (TTMP) are commercially available as pure substances and declared as natural flavoring agents [3].

As shown in Table 1, the total volatiles showed a gradual increase during incubation time up to 12 days (38.21 × 10^6^) followed by a significant decrease at 15 days incubation (8.89 × 10^6^). These results confirm those of odor sensory evaluation (Fig. 3a). Acetoin, the biodegradation product of sugar [7], showed a significant decrease during incubation period, that could confirm its role as a precursor of pyrazines.

Dickschat et al. [6] proposed the pathway for the biosynthesis of TTMP from acetoin in *Carynebacterium glutamicum*. Aectoin might oxidized to butandione by acetoin dehydrogenase (AD) and transaminated to 3-aminobutanone. Two units of this compound were condensed to produce tetramethyldihydropyrazine which spontaneously oxidized to TTMP. Two pathways were proposed for the biosynthesis of TMP, the first required one unit of acetoin and C2 building block such as glycolaldehyde or glycosal. While the second required acetoin and a C3 unit such as hydroxyacetone or methylglyoxal [6, 10]. The biosynthesis of 2-methylpyrazine and 2, 5-dimethylpyrazine required C2 + C3 and C3 + C3, respectively. The two compounds may be biosynthesised from the respective C3 unit and the acetoin derivative 3-hydroxy-pentane-2-one or 2-hydroxypentane-3-one.

The decrease in the total content of pyrazines at the end of incubation time (15 days) may be correlated to the fact that some microorganisms used the alkylated pyrazines as a sole carbon, nitrogen and energy source [29] under aerobic conditions. The significant decrease in total pyrazines content at 15 days may be correlated to the decrease in the amount of their precursors in addition to the strong antifungal activity of 2,5-dimethylpyrazine [30], that showed a significant increase at 15 days incubation (Table 1). There is apparent lack of data concerning the mechanisms of alkylated pyrazines formation and degradation by fungi. Also for bacteria, most of the hypothetical pathways for pyrazines degradation were correlated to their disappearance in cultures isolated bacteria [24]. Fungi from South Africa were screened using sensory evaluation for the presence nutty aroma, that indicates the ability of the fungi to produce pyrazines [31]. *Aspergillus* and *Penicillum* species were the most flavour procedures. The ability of *Penicillum* species to produce 2-methoxy-3-isobutylpyrazines was confirmed *via* sensory and chemical analysis [31]. Fengqing et al [32] isolated three new pyrazine derivatives (talaropyrazines) from *Talaromyces minioluteus* (*Penicillium minioluteum*). The results of nuclear magnetic resonance (NMR) and high performance liquid chromatography (HPLC) analysis allowed the authors to propose a general mechanism for their formation. Recently, the presence of 2,5-dimethylpyrazine in the headspace of *Aspergillus Niger* culture was confirmed by GC-MS analysis.

As shown in Table 1 the highest yield of the biosynthesized pyrazines was obtained from culture incubated for 12 days. Therefore, the effect of varying the initial pH of the medium on the pyrazines production after incubation for 12 days was studied. The initial pH of the medium was adjusted at pH 6, 7, 8, and 9. The results revealed that the optimum pyrazines production was at initial pH 8. A positive correlation was found between the total volatiles (Table 1) and odor sensory analysis. Leelawiwat et al. [9] optimized the factors that affect on the production of pyrazines from *Bacillus* sp culture and found that the pyrazines were produced more efficiently at pH 8.5.

The effect of enrichment of the culture medium with amino acids (L-threonine, lysine and serine) on the bioproduction of pyrazines was investigated. As shown in Fig. 3c the total yield of pyrazines showed a slight increase by addition of 0.5 g amino acids/50 mL medium followed by a significant decrease (*P* < 0.05) by addition of 1, 1.5, and 2 g amino acids/50 mL medium.

L-threonine-3-dehydrogenase (TDH) catalyze the reaction from L-threonine to L-2-amino-acetoacetate that can be decarboxylated spontaneously to form aminoacetone followed by cyclization and oxidation to form 2,5-dimethylpyrazine. Among eight microorganisms the fungal genus, *Aspergillus*, was found to have TDH enzyme [33] that could confirm its ability to produce the alkylated pyrazines. The significant decrease in the total volatiles by increasing the concentration of the added amino acids may be correlated to the decrease in pH of the medium during amino acid catabolism [34].

### Encapsulation effect on the flavor quality

The aforementioned results revealed that the volatiles generated from *Tolypocladium inflatum* SRH81 culture, incubated for 12 days at pH 8.0 and enriched with 0.5 g amino acids/50 mL medium, showed the best results. So, it was selected and subjected to encapsulation in gum Arabic by a spray drier. As shown in Fig. 3d, encapsulation resulted in a slight decrease in the aroma intensity. The GC-MS analysis revealed a significant decrease in the total content of pyrazines. This finding may be correlated to the hypothesis that high temperature (150 °C) and presence of oxygen catalyze the dehydration and oxidation reaction during encapsulation process and thus gave rise to the decrease in content of the encapsulated compounds [10, 35]. Figure 3 revealed a quit agreement with the results of odor sensory analysis and those of GC-MS analysis. The insignificant decrease in the flavor intensity may be correlated to the increase in the content of the low volatile compounds such as TTMP at the expense of the high volatile compounds [10, 28].

## Conclusions

Roasted-nutty flavor is one of the most popular flavors for consumers. Alkylated pyrazines are responsible for the roasted-nutty aroma, so they have a great importance in food industry. Currently, consumers dissatisfied with chemical flavors and at the same time, extracting the flavor from natural sources is difficult and expensive, so using microorganisms to produce the flavor is an economical safe alternative. *Tolypocladium inflatum* SRH81 was the only producer of roasted–nutty aroma among nine screened species of fungi isolated from the Egyptian soil. Culture conditions (incubation time, pH value and supplementation with amino acids) were optimized to obtain the highest aroma production. The results revealed that incubation for 12 days at pH 8 and supplementation of the culture with 0.5 g amino acids/50 mL based medium were the appropriate conditions for the high production of pyrazines and best aroma quality. The results of GC-MS analysis of the headspace volatiles of each culture confirmed those of odour sensory analysis. Encapsulation, in addition to converting the liquid roasted-nutty flavour into easy handle powder, showed insignificant effect on aroma quality.

## Supplementary Information

**Additional file 1: Table S1.** Parameters affect the culture growth and pyrazines production.

## Data Availability

The data generated or analyzed during this study are available from the corresponding author on reasonable request.

## Abbreviations

HS-SPME: Headspace-solid phase microextraction
GC-MS: Gas chromatography-mass spectrometry
FAD: Food and Drug Administration
EFSA: European Food safe Authority
MA: Malt extract agar
DNS: Dinitrosalicylic acid
DVB/CAR/PDMS: Divinylbenzene/carboxe/polydimethylsiloxane
TIC: Total ion chromatograms
R-N: Roasted-nutty flavor
E-R-N: Encapsulated roasty-nutty flavor
TMP: 2, 3, 5-trimethylpyrazine
2, 5-DMP: 2, 5-dimethylpyrazine
2,6-DMP: 2,6-dimethylpyrazine
TTMP: 2, 3, 5, 6-tetramethylpyrazine
AD: Acetoin dehydrogenase
TDH: Threonine-3-dehydrogenase
DM: Dry matter
CS: Consumed sugar
KI: Retention indices
