# Supplementary material for: Optimization of the production of roasted-nutty aroma by a newly isolated fungus Tolypocladium inflatum SRH81 and impact of encapsulation on its quality
Source: J Genet Eng Biotechnol. 2022 Nov 23;20:159. doi: 10.1186/s43141-022-00445-x (PMC9684363; doi:10.1186/s43141-022-00445-x)
Supplement: Supplementary file 1 — Additional file 1: Table S1. Parameters affect the culture growth and pyrazines production. [file 43141_2022_445_MOESM1_ESM.docx]

**Table 1S Parameters affect the culture growth and pyrazines production**

| Amino acid | pH | Time (days) |
| --- | --- | --- |
| 0.0 | 7 | 6 |
| 0.0 | 7 | 9 |
| 0.0 | 7 | 12 |
| 0.0 | 7 | 15 |
|  |  |  |
| 0.0 | 6 | 12 |
| 0.0 | 7 | 12 |
| 0.0 | 8 | 12 |
| 0.0 | 9 | 12 |
|  |  |  |
| 0.5 | 8 | 12 |
| 1.0 | 8 | 12 |
| 1.5 | 8 | 12 |
| 2.0 | 8 | 12 |
